# M6A-dependent RNA condensation underlies FUS autoregulation and can be harnessed for ALS therapy development

**DOI:** 10.1126/sciadv.adx1357

**Published:** 2025-07-23

**Authors:** Wan-Ping Huang, Vedanth Kumar, Karen Yap, Haiyan An, Sabin J. John, Rachel E. Hodgson, Anna Sanchez Avila, Emily Day, Brittany C. S. Ellis, Tek Hong Chung, Jenny Lord, Michaela Müller-McNicoll, Eugene V. Makeyev, Tatyana A. Shelkovnikova

**Affiliations:** ^1^ Sheffield Institute for Translational Neuroscience (SITraN) and Neuroscience Institute, University of Sheffield, Sheffield, UK.; ^2^ Centre for Developmental Biology, King’s College London, London, UK.; ^3^ Medical School, Swansea University, Swansea, UK.; ^4^ Institute of Molecular Biosciences, Goethe University, Frankfurt am Main, Germany.

## Abstract

Mutations in the *FUS* gene cause aggressive amyotrophic lateral sclerosis (ALS-FUS). Beyond mRNA, *FUS* generates partially processed transcripts retaining introns 6 and 7. We demonstrate that these FUSint6&7-RNA molecules form nuclear condensates, scaffolded by the highly structured intron 7 and associated with nuclear speckles. Using hybridization-proximity labeling proteomics, we show that the FUSint6&7-RNA condensates are enriched for splicing factors and the N6-methyladenosine (m6A) reader YTHDC1. These ribonucleoprotein structures facilitate posttranscriptional FUS splicing and depend on m6A/YTHDC1 for integrity. In cells expressing mutant FUS, FUSint6&7-RNAs become hypermethylated, which in turn stimulates their condensation and splicing. We further show that FUS protein is repelled by m6A. Thus, ALS-FUS mutations may cause abnormal activation of FUS posttranscriptional splicing through altered RNA methylation. Notably, ectopic expression of FUS intron 7 sequences dissolves endogenous FUSint6&7-RNA condensates, down-regulating FUS mRNA and protein. Our findings reveal a condensation-dependent mechanism regulating FUS splicing, with possible therapeutic implications for ALS.

## Teaser

M6A-driven condensation of an intron-containing FUS RNA isoform regulates its splicing and has translational potential in ALS.

## INTRODUCTION

FUS is an abundant RNA binding protein (RBP) with numerous roles in cellular RNA metabolism (*1*). The *FUS* gene located on chromosome 16 encodes a 526–amino acid protein. Since the discovery of FUS’s association with amyotrophic lateral sclerosis (ALS) in 2009 (*2*, *3*), >50 ALS-FUS mutations have been identified, most of which are missense mutations [reviewed in (*4*)]. Although *FUS* mutations account for only 4% of familial ALS, they are the most frequent cause of juvenile ALS—a particularly aggressive form of the disease (*5*–*7*). Most *FUS* mutations map to its C terminus and lead to the impairment or complete loss of the nuclear localization signal (NLS) (*4*). FUS cytoplasmic mislocalization likely triggers a combination of loss- and gain-of-function mechanisms; however, their relative contribution to ALS pathology is still debated. Studies in mice have demonstrated that mutant FUS expression, but not FUS knockout (KO), is sufficient to cause neurodegeneration (*8*–*12*), suggesting that FUS toxic gain of function plays a key role in the disease. This mechanism is further supported by the effects of a recently developed antisense oligonucleotide (ASO) therapy, where simultaneous depletion of both mutant and normal FUS inhibited neurodegeneration in mice and potentially in humans (*9*).

FUS accumulation in large cytoplasmic inclusions in ALS-FUS postmortem tissue (*13*–*15*), ALS mutations in *FUS* 3′ untranslated region leading to its overexpression (*16*), and FUS mRNA up-regulation in physiological cell models of ALS-FUS (*17*) all point to a disrupted control over its cellular levels as a disease hallmark. One reported mechanism supports changes in autoregulation, where FUS protein suppresses normal splicing of its pre-mRNA by promoting exon 7 skipping and nonsense mediated decay (NMD) (*18*). A more recent study showed that introns 6 and 7 are often retained in FUS pre-mRNA (*19*). These relatively long, highly conserved introns have multiple FUS protein binding sites, raising a possibility that FUS promotes their retention, thereby reducing the translatable FUS mRNA pool via a negative-feedback mechanism. These introns were found to be spliced more efficiently in in vitro and in vivo models of ALS-FUS (*19*). Retention of FUS introns 6 and 7 was enhanced in a mouse ALS-FUS model upon introduction of the full human *FUS* transgene and was associated with a marked rescue of the disease phenotype (*20*).

Modulation of disrupted FUS autoregulation may provide an attractive therapeutic strategy, bypassing the undesirable FUS loss-of-function effects of the ASO treatment. Here, we report that FUS transcripts with retained introns 6 and 7 (FUSint6&7-RNA thereafter) form a ribonucleoprotein (RNP) condensate in the nucleus. Compositional analysis of these structures by a hybridization-proximity labeling approach revealed enrichment of proteins involved in RNA splicing and regulation by N6-methyladenosine (m6A) modification. We provide evidence that ALS-linked FUS mutations promote m6A methylation of FUS transcripts, which in turn stimulates their nuclear condensation and enhances the production of fully spliced FUS mRNA. Notably, introduction of exogenous FUS intron 7 sequences resulted in disrupted condensation of endogenous FUSint6&7-RNA and suppressed (mutant) FUS mRNA and protein production. Our study advances current understanding of FUS expression regulation and describes an approach to the modulation of FUS levels in the disease context.

## RESULTS

### Regulation of FUS RNA with retained introns 6 and 7 in WT and ALS-FUS cells

**Fig. 1.**
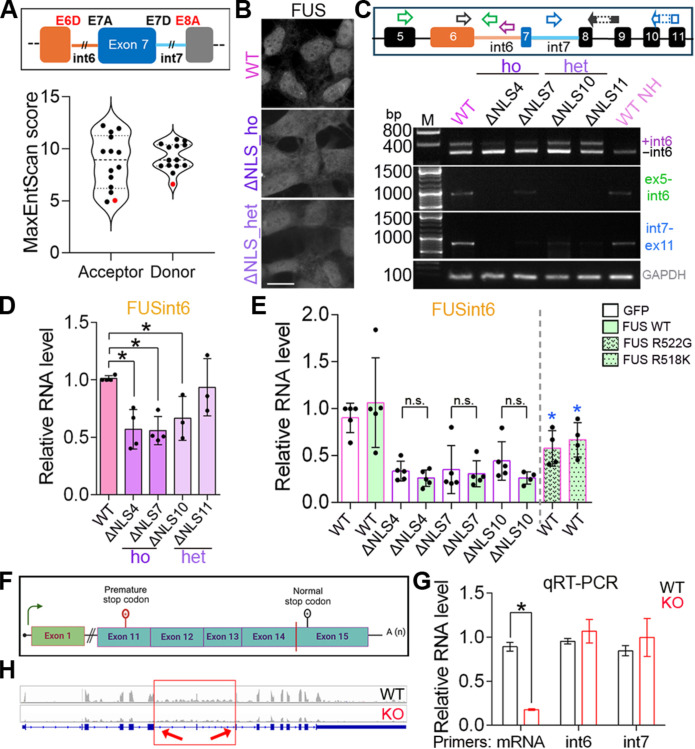
Regulation of FUS RNA with retained introns 6 and 7. (**A**) Splice sites flanking the retained region in FUSint6&7-RNA are weaker compared to other splice sites in the *FUS* gene. MaxEntScore was used to determine the splice site strength. All splice sites are plotted, and E6D and E8A are given in red. (**B**) FUS mislocalization in FUSΔNLS cell lines used in the study. ho, homozygous; het, heterozygous. (**C** and **D**) Reduced retention of introns 6 and 7 in FUS RNA demonstrated by PCR (C) and qRT-PCR (D). WT NH–WT cell samples not heated (NH) during RNA extraction. A combination of three primers, FUS_ex6_for, FUS_int6_rev, and FUS_ex8/9_rev, was used to detect intron 6 inclusion. *P < 0.05, *N* = 3 to 4, Kruskal-Wallis with Dunn’s post hoc test. M, DNA molecular weight marker. (**E**) FUS overexpression does not restore FUS intron 6 retention in FUSΔNLS lines, whereas expression of mutant FUS down-regulates FUSint6&7-RNA in WT cells. GFP-tagged FUS and its ALS-linked variants R522G (mainly cytoplasmic) and R518K (mainly nuclear) were used. *N* = 3 to 5. *P < 0.05, Mann-Whitney *U* test, as compared to WT cells expressing WT FUS-GFP. n.s., not significant. (**F** to **H**) FUSint6&7-RNA level remains unchanged in FUS KO cells with intact FUS transcription but undetectable FUS protein. *FUS* locus CRISPR-Cas9 editing schematic (F), FUSint6&7-RNA analysis by qRT-PCR (G), and RNA sequencing (H) are shown. *P < 0.05, *N* = 3, Mann-Whitney *U* test.

We first investigated the molecular basis for retention of FUS introns 6 and 7. Analysis of exon 7–spanning reads in mRNA sequencing data from the S3 clone of HeLa cells (*21*) and from human motor neurons (*22*) revealed that these two introns are retained in a mutually dependent manner (fig. S1). Although the length and the GC content of introns 6 and 7 are typical of internal introns in multi-intron transcripts (fig. S2, A and B), their core cis-splicing elements deviate markedly from the genome-wide medians. In particular, the strength of the exon 6/intron 6 donor site (5′ss), as measured by maximum entropy modeling (MaxEntScan) analysis (*23*), is relatively low (17.8th percentile; fig. S2C). The intron 7/exon 8 acceptor site (3′ss) is likewise weak (10.4th percentile; fig. S2D). Moreover, the predicted branch point of intron 7 lies unusually far from the 3′ss (97.2nd percentile; fig. S2E), with a correspondingly wide AG dinucleotide exclusion zone (*24*, *25*). The weakness of the exon 6/intron 6 donor and the intron 7/exon 8 acceptor was also evident when compared with other splice sites within the *FUS* gene (Fig. 1A and table S1). These observations suggest a possible mechanism for the relatively inefficient splicing of the intron 6–exon 7–intron 7 region. Of note, RBPmap (*26*) did not reveal notable differences in the repertoire of RBP binding sites in FUS introns 6 and 7 distinguishing them from the rest of *FUS* introns.

Consistent with the findings in murine central nervous system tissue (*19*), a reduction in FUSint6&7-RNA was observed in human ALS-FUS cell models—FUSΔNLS SH-SY5Y (human neuroblastoma) lines generated by CRISPR-Cas9 editing to endogenously express FUS lacking NLS (Fig. 1, B to D) (*17*). Unexpectedly, we found that this reduction cannot be rescued by overexpression of green fluorescent protein (GFP)–tagged wild-type (WT) FUS (Fig. 1E). Furthermore, ectopic expression of ALS-FUS mutants, R522G or R518K, significantly down-regulated FUSint6&7-RNA in WT neuroblastoma cells (Fig. 1E). This suggested that a mutant FUS gain-of-function rather than a loss-of-function mechanism is responsible for reduced retention of FUS introns 6 and 7 in ALS-FUS models. To address this directly, we used a previously generated FUS KO SH-SY5Y line (*17*) that has a premature stop codon in the *FUS* gene introduced by CRISPR-Cas9 editing, leading to FUS mRNA degradation by NMD (Fig. 1F). This cell line expresses normal levels of FUS pre-mRNA but has severely reduced FUS mRNA (to ~15% of normal) and undetectable protein expression (Fig. 1G) (*17*). We found that the FUSint6&7-RNA level was not affected in this cell line compared to WT SH-SY5Y cells—demonstrated by quantitative reverse transcription polymerase chain reaction (qRT-PCR) (Fig. 1G) and RNA sequencing [dataset from (*17*)] (Fig. 1H). These results reveal the importance of a gain-of-function mechanism in affecting the abundance of FUS transcripts with retained introns 6 and 7 in mutant FUS-expressing cells.

### FUSint6&7-RNA molecules form multimolecular foci in the nucleus

**Fig. 2.**
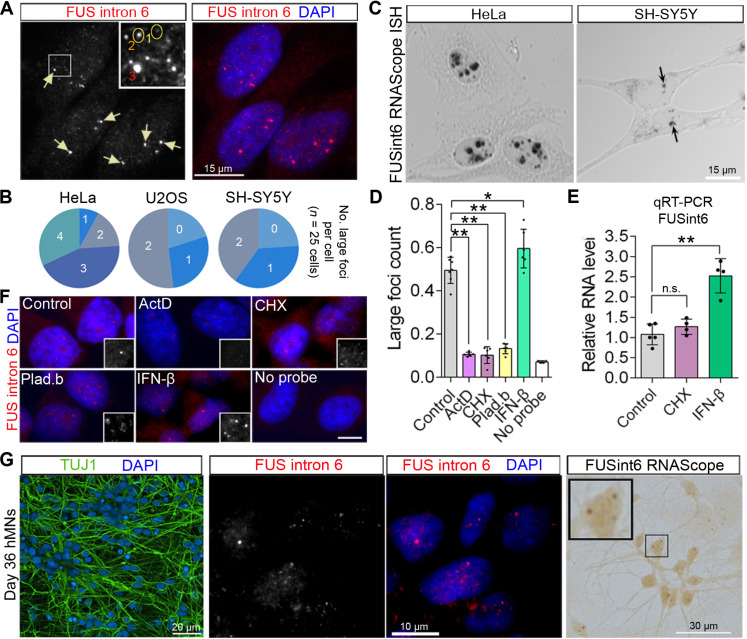
FUS RNA with retained introns forms dynamic nuclear foci. (**A**) FUSint6&7-RNA forms nuclear foci. Representative images for RNA-FISH with a FUS intron 6–specific probe in HeLa cells are shown. Three types of foci, based on their size, are labeled. Arrows indicate the large (type 3) foci, which likely assemble near the sites of transcription. (**B**) Large foci (type 3) frequency per cell in different cell lines corresponds to the cell line ploidy. (**C**) FUSint6&7-RNA foci visualized using a FUS intron 6 RNAscope-ISH probe with chromogenic detection. Representative images for HeLa and SH-SY5Y cells are shown. (**D** to **F**) FUSint6&7-RNA foci formation relies on ongoing transcription and is sensitive to changes in RNA metabolism. HeLa cells were treated with actinomycin D (actD), CHX, or pladienolide B (plad.b) for 4 hours or with IFN-β for 24 hours. FUSint6-positive foci were quantified by a high-content imaging assay (D) and analyzed by qRT-PCR (E) and by high-resolution imaging (F). In (D), data are for six individual wells, from a representative experiment, *P < 0.05 and **P < 0.01, Kruskal-Wallis with Dunn’s test. In (E), *N* = 4 to 5, **P < 0.01, Kruskal-Wallis with Dunn’s test. (**G**) FUSint6&7-RNA foci form in cultured human motor neurons. Day 36 neurons were used for RNA-FISH and RNAscope-ISH (FUS intron 6–specific probes). DAPI, 4′,6-diamidino-2-phenylindole.

Given that the FUSint6&7-RNA species accumulate in the nucleus (*19*), we aimed to characterize their fate and regulation in this compartment. We previously showed that a nuclear-localized transcript with retained introns produced from the mouse *Srsf7* gene can form phase-separated granules (*27*). Using an RNA–fluorescence in situ hybridization (FISH) probe pool covering FUS intron 6 (37 oligonucleotides), bright nuclear foci were detected in all widely used cell lines (HeLa, U2OS, SH-SY5Y, and human fibroblasts) (Fig. 2A and fig. S3A).

In addition to these larger granules, multiple smaller foci were also detectable in the nucleoplasm (Fig. 2A, inset). The smallest/dimmest foci (“1” in Fig. 2A, inset) presumably corresponded to single RNA molecules, whereas the intermediate-size foci (“2”) represented their clusters, and the largest structures (“3”) likely corresponded to a build-up of FUSint6&7-RNA at the sites of its transcription. The number of large foci per cell matched the cell line ploidy (e.g., three to four in hypertriploid—3n + HeLa cells and two foci in diploid U2OS and SH-SY5Y cells) (Fig. 2B). The large foci were not composed of nascent or uniformly unspliced FUS pre-mRNAs since they were not recognized by a FUS intron 1–specific probe pool (fig. S3A). FUSint6&7-RNA foci were also readily detectable using RNAscope-ISH with chromogenic detection (Fig. 2C). Notably, this detection approach revealed a notably larger granule size in HeLa cells as compared to SH-SY5Y cells, pointing to variable numbers of molecules per focus in different cell lines. A FUS intron 7–specific probe pool (33 oligonucleotides) also detected characteristic foci in HeLa cells (fig. S3B). In subsequent experiments, either FUSint6 or -7 specific probes were used to detect endogenous FUSint6&7-RNA foci.

Using an automated foci imaging and quantification assay on Opera Phenix high-content imaging system (fig. S3C), large FUSint6&7-RNA foci were found to be dispersed by actinomycin D, cychoheximide (CHX), and a splicing inhibitor pladienolide B (Fig. 2D). In contrast, interferon-β (IFN-β)—previously shown to up-regulate FUS at the RNA level (*28*)—increased the number of foci (Fig. 2D). CHX, which is known to inhibit NMD, had no effect on FUSint6&7-RNA abundance, in line with the previous report (*19*), whereas IFN-β increased it (Fig. 2E). CHX and pladienolide B induced fragmentation of the large foci (type 3) into smaller dots, without changes to the total signal intensity (Fig. 2F). This suggested that factors other than FUSint6&7-RNA itself are required for maintaining the foci integrity.

Last, we confirmed that nuclear FUSint6&7-RNA granules form in an ALS-relevant cell type, human motor neurons (Fig. 2G). Similar to non-neuronal cells, FUSint6&7-RNA expression remained unchanged in response to CHX and was up-regulated by IFN-β (fig. S3D). Thus, partially processed FUS transcripts can assemble dynamic multimolecular structures in the nuclei of diverse cell types.

### FUSint6&7-RNA foci are condensates scaffolded by intron 7

**Fig. 3.**
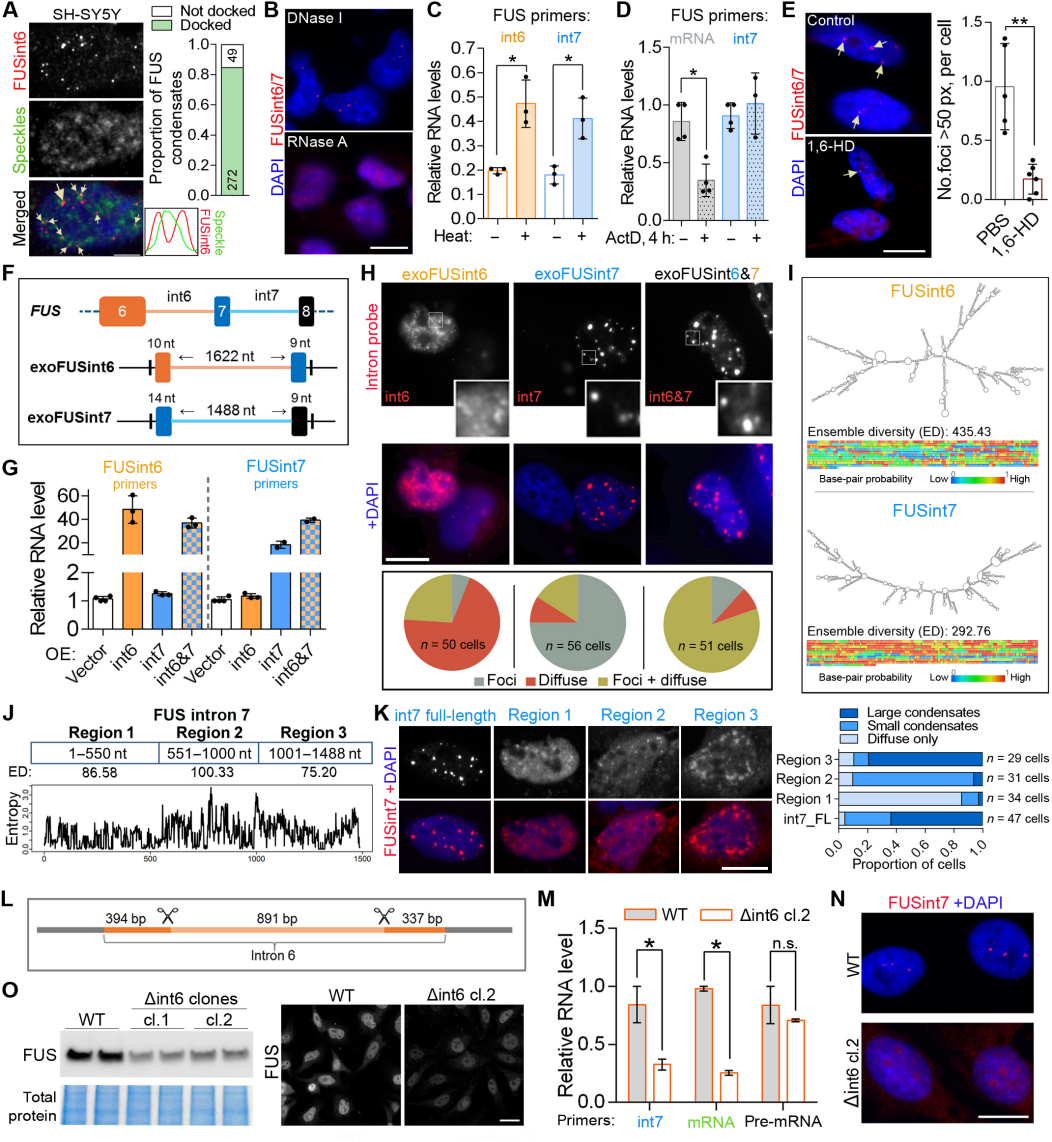
FUSint6&7-RNA foci are phase-separated condensates nucleated by intron 7 and associated with splicing speckles. (**A**) FUSint6&7-RNA foci are associated with splicing speckles. PNN was a speckle marker; *n* = 20 cells. (**B**) RNase, but not DNase, treatment eliminates FUSint6&7-RNA foci. (**C**) FUSint6&7-RNA is semiextractable. qRT-PCR was performed in samples with or without heating before RNA isolation. *N* = 3, *P < 0.05, Mann-Whitney *U* test. (**D**) FUSint6&7-RNA is relatively stable. FUSint6&7-RNA and FUS mRNA levels were analyzed by qRT-PCR after a 4-hour actinomycin D treatment. GAPDH was used for normalization. *N* = 3 to 4, *P < 0.05, Mann-Whitney *U* test. h, hours. (**E**) FUSint6&7-RNA foci are sensitive to an LLPS-disrupting agent. 1,6-Hexanediol (1,6-HD) treatment was performed in semipermeabilized cells. Five to six fields of view were analyzed from a representative experiment; **P < 0.01, Mann-Whitney *U* test. (**F** and **G**) Comparable expression of exogenous FUS introns. Construct schematics (F) and qRT-PCR analysis (G) are shown. *N* = 3 to 4. OE, overexpression. (**H**) FUS intron 7, but not intron 6, forms compact, dense nuclear condensates upon ectopic expression. (**I**) FUS intron 7 is more structured than intron 6, with lower ensemble diversity. Minimum free energy structure and base-pairing probabilities heatmaps were generated by RNAfold. (**J** and **K**) Molecular dissection of FUS intron 7 condensation. Full-length (FL) intron without splice sites was used as a control. Regions analyzed (J) and representative images and quantification (K) are shown. (**L** to **O**) CRISPR-mediated deletion of a middle portion of FUS intron 6 destabilizes FUSint6&7-RNA and leads to FUS mRNA and protein depletion. Positions of sgRNAs (L), levels of FUS RNA species (M), FUSint6&7-RNA condensate analysis (N), and FUS protein levels (O) in homozygous Δint6 clones (cl.1 and 2) are shown. *N* = 3 to 4, *P < 0.05, Mann-Whitney *U* test. SH-SY5Y was used in (A) and HeLa in other panels. Scale bars, 2 μm in (A); 10 μm in [(B), (E), (H), (K), and (N)]; and 20 μm in (O).

We next focused on detailed characterization of the FUSint6&7-RNA foci. Both large and small foci were typically (>80%) present on the border of, or “docked” to, nuclear speckles (Fig. 3A and fig. S4). This pattern resembled that of paraspeckles, which are nuclear bodies assembled by long noncoding RNA NEAT1_2, that both cluster at the transcription site and distribute throughout the nucleus, becoming associated with speckles (*29*).

Ribonuclease A (RNase A) treatment in semipermeabilized cells eliminated the large FUSint6&7-RNA foci, whereas deoxyribonuclease I (DNase I) treatment had no effect (Fig. 3B). Architectural RNAs (arcRNAs) that assemble phase-separated nuclear bodies are tightly packed in RNPs, such that heating or mechanical shearing is required for their efficient isolation during RNA purification with a TRIzol-type reagent (*30*). FUSint6&7-RNA had semiextractable properties; when the heating step was omitted (included into our standard RNA isolation protocol, see Materials and Methods), the yield of this RNA was significantly decreased (Fig. 3C). Although transcription block prevents granule formation by arcRNAs (*30*), the RNA itself can remain stable for hours, for example, as in the case of NEAT1_2 (*31*). We found that the FUSint6&7-RNA level remains unchanged after a 4-hour actinomycin D treatment [when normalized to GAPDH that has a half-life of ~8 hours (*32*)], whereas the FUS mRNA level was decreased to ~25% at this time point (Fig. 3D). Condensates formed by liquid-liquid phase separation (LLPS) are sensitive to 1,6-hexanediol (1,6-HD)—an aliphatic alcohol that disrupts weak electrostatic interactions (*33*). Short 1,6-HD treatment in semipermeabilized cells decreased the number of FUSint6&7-RNA foci (Fig. 3E).

We next examined the relative contribution of introns 6 and 7 to FUSint6&7-RNA condensate assembly. Constructs for ectopic expression of the two introns (exoFUSint6 and -7 thereafter) were generated, by cloning the full intronic sequence with the splice sites into an expression vector (Fig. 3F). qRT-PCR confirmed accumulation of both introns when expressed separately or coexpressed (Fig. 3G). RNA-FISH and nuclear-cytoplasmic fractionation demonstrated that although both overexpressed introns were largely retained in the nucleus, exoFUSint7 readily formed dense foci, whereas exoFUSint6 remained diffuse (albeit we cannot exclude the formation of small condensates masked by the diffuse signal) (Fig. 3H and fig. S5A). FUS intron 1, which has a similar length (2170 nt) but is not retained, also remained diffuse when overexpressed (fig. S5B). Nuclear foci formed by exoFUSint7 did not overlap with known nuclear bodies—paraspeckles (NEAT1_2), Cajal bodies (coilin p80), or Gems (SMN) (fig. S5C). Furthermore, exoFUSint7 condensates responded to CHX and pladienolide B treatments in a way similar to their endogenous counterparts (fig. S5D). RNAfold predictions (*34*) indicated that intron 7 is more structured as compared to intron 6, with lower positional entropy, higher probability of base pairing, and lower ensemble diversity (Fig. 3I).

To understand the contribution of specific portions of FUS intron 7 to its condensation behavior, we first analyzed its 5′-proximal (region 1; 1 to 550 nt), middle (region 2; 551 to 1000 nt), and 3′-proximal (region 3; 1001 to 1488 nt) segments using RNAfold (Fig. 3J). On the basis of positional entropy and ensemble diversity scores, region 3 exhibited the highest propensity to form secondary structures, followed by region 1 and then region 2 (Fig. 3J). To experimentally assess the condensation properties of these regions, we generated three corresponding expression constructs, along with a construct encoding the full-length intron 7 lacking splice sites. Notably, transcripts produced from this full-length construct without splice sites efficiently formed condensates (Fig. 3K), suggesting that intron 7 condensation is likely independent of spliceosome assembly. We next visualized the cellular localization patterns of these three individual intron 7 regions using the same FUS intron 7 RNA-FISH probe set—with 14, 8, and 11 oligonucleotide probes mapping to regions 1, 2, and 3, respectively. None of the fragments alone was sufficient to form the large, compact condensates characteristic of full-length intron 7 (Fig. 3K). Region 1 transcripts showed predominantly diffuse nuclear localization with occasional small condensates. Region 2 transcripts tended to form small nuclear condensates, often against a diffuse background. Last, region 3 transcripts formed large but amorphous and less dense nuclear condensates (Fig. 3K). Region 2 and region 3 transcripts were partially redistributed to the cytoplasm. These results indicate that FUS intron 7 condensation depends on a combination of RNA elements distributed along its length.

To establish the contribution of the two introns to FUSint6&7-RNA condensate assembly at the endogenous level, we targeted FUS introns 6 and 7 using specific CRISPR-Cas9 single guide RNAs (sgRNAs). While we were unable to obtain viable clones lacking intron 7 sequences, we successfully generated HeLa cells with a large portion of intron 6–deleted Δint6 clones (Fig. 3L and fig. S6). FUSint6&7-RNA (measured with an intron 7–specific primer pair) was significantly down-regulated in these clones (Fig. 3L). This was accompanied by the loss of FUSint6&7-RNA condensates (Fig. 3M) and reduced expression of FUS mRNA and protein (Fig. 3, N and O). Notably, the mutation had no detectable effect on *FUS* pre-mRNA levels as measured by qRT-PCR with intron 1–specific primers (Fig. 3L).

These data indicate that the foci containing multiple copies of FUSint6&7-RNA exhibit the properties of biomolecular condensates. Their assembly appears to depend on the structured intron 7 and LLPS. Intron 6, on the other hand, may be required for their proper processing and/or stability.

### FUSint6&7-RNA condensates are positive regulators of FUS expression

**Fig. 4.**
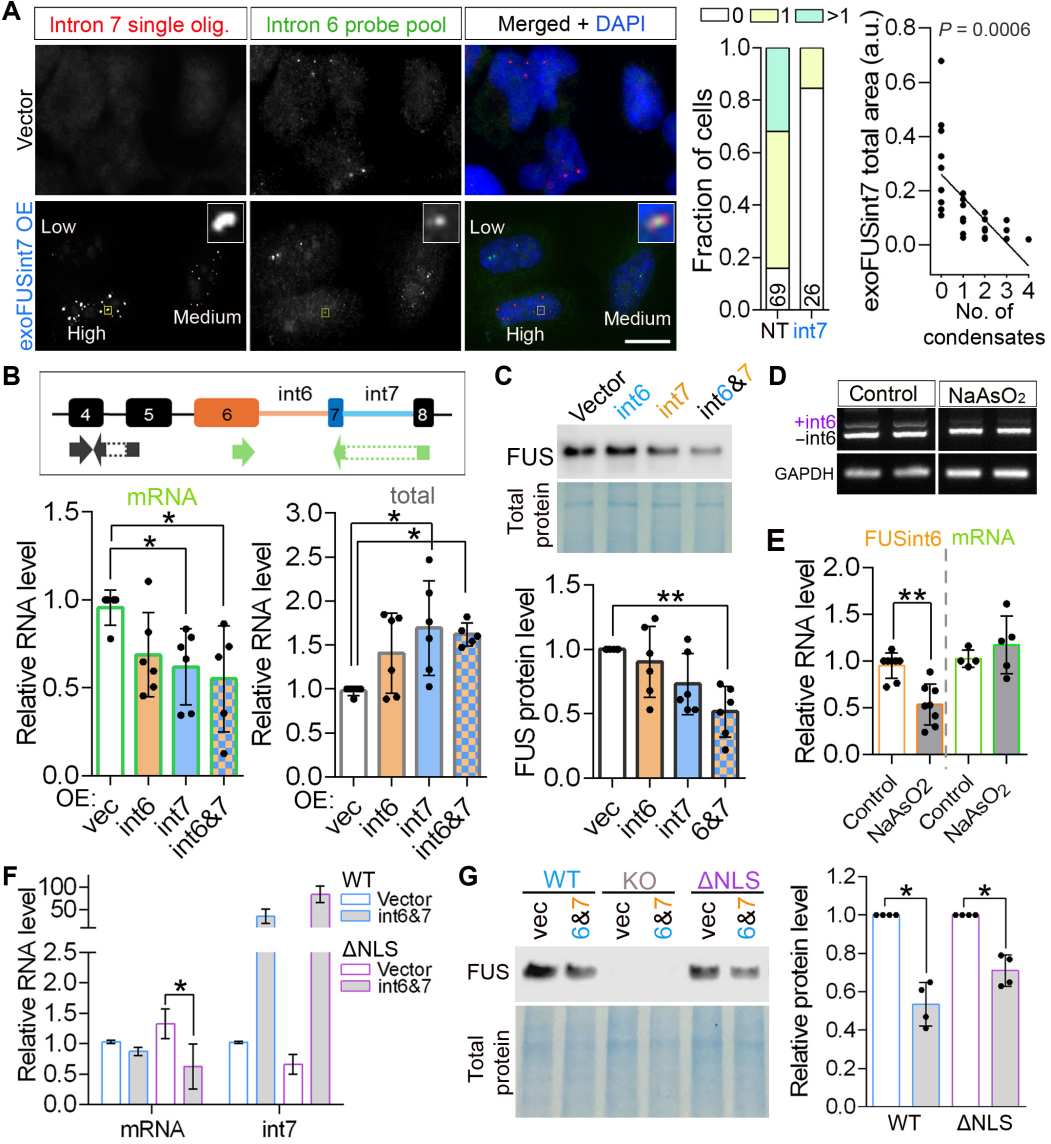
FUSint6&7-RNA condensates regulate FUS mRNA levels. (**A**) Ectopic expression of FUS intron 7 dissolves endogenous FUSint6&7-RNA condensates. Single FUSint7 oligonucleotide probe recognizing exoFUSint7 condensates but not endogenous FUSint6&7-RNA condensates was used in combination with the Stellaris FUSint6 probe pool. Representative images and quantification of endogenous FUSint6&7-RNA condensates are shown. Cells with high, medium, and low exoFUSint7 expression are indicated. Inset shows fusion of a FUSint6&7-RNA condensate with an exoFUSint7 condensate. Number of endogenous condensates quantified in exoFUSint7 condensate–containing cells (int7) versus nontransfected cells (NT) in the same field of view (FOV) is indicated inside the bars. Correlation between the area of exoFUSint7 signal in individual nuclei and the number of endogenous FUSint6&7-RNA condensates are also shown (25 cells). Scale bar, 10 μm. (**B**) Ectopic expression of exoFUSint7 or exoFUSint6 + 7 leads to FUS mRNA down-regulation. qRT-PCR analysis of FUS total (FUS mRNA + FUSint6&7-RNA) and mRNA levels is shown. *N* = 3 to 5, *P < 0.05, Kruskal-Wallis with Dunn’s test. (**C**) Ectopic expression of exoFUSint6 + 7 leads to FUS protein down-regulation. Representative Western blot and quantification for HeLa cells are shown. *N* = 6, **P < 0.01, Kruskal-Wallis with Dunn’s test. (**D** and **E**) FUS intron retention is responsive to cellular stress. FUS RNA levels were analyzed in cells recovering from NaAsO_2_ stress (1-hour stress + 3-hour recovery) by PCR with a triple primer combination (D) and qRT-PCR (E). *N* = 5 to 7, **P < 0.01, Mann-Whitney *U* test. (**F** and **G**) ExoFUSint7 expression down-regulates FUS mRNA and protein in an ALS-FUS cell model. FUSΔNLS lines were analyzed by qRT-PCR (F) and Western blot (G). *N* = 3 to 4, *P < 0.05, Mann-Whitney *U* test. In (F) and (G), ΔNLS10 and ΔNLS4 lines were used, respectively. In (F), data for intron 7–specific primer were included to confirm successful exoFUSint7 overexpression. a.u., arbitrary units; vec, vector.

Since FUS intron 7 in isolation can assemble condensates, we hypothesized that overexpressing intron 7 sequences may disrupt endogenous FUSint6&7-RNA condensates due to sequestration of their essential components. To test this prediction, we used a combination of the FUSint6-specific RNA probe pool (Stellaris)—for detection of the endogenous FUSint6&7-RNA—and a single FUSint7-specific oligonucleotide probe—for detection of exoFUSint7 condensates. This FUSint7 probe had low labeling efficiency and detected exoFUSint7 condensates but not the endogenous FUSint6&7-RNA condensates (Fig. 4A). With this approach, we found that FUSint6&7-RNA condensates were dissolved in most cells that developed exoFUSint7 de novo condensates (Fig. 4A). We also observed fusion events between the endogenous and exogenous condensates (Fig. 4A, inset).

Analysis of FUS mRNA levels in cells expressing exoFUSint7, separately or in combination with exoFUSint6, demonstrated that this modification triggers a significant down-regulation of FUS mRNA (by ~30%; Fig. 4B). This was accompanied by an increase in the total FUS transcript levels (FUS mRNA + FUSint6&7-RNA) (Fig. 4B), due to up-regulation of endogenous FUSint6&7-RNA (detected using intron 6–specific primers) (fig. S7, A and B). exoFUSint6 alone also up-regulated FUSint6&7-RNA (fig. S7A); however, it did not significantly change the abundance of FUS mRNA (Fig. 4A). This milder effect is consistent with the limited condensate-forming ability of intron 6. The FUS protein level was decreased in exoFUSint6 + 7–expressing cells, mirroring its mRNA down-regulation (Fig. 4C). FUS intron 1 overexpression did not affect FUS mRNA levels (fig. S7C). These data suggested that FUSint6&7-RNA condensates may represent reservoirs of FUS transcripts poised for FUS mRNA production, whose disruption has a suppressive effect on the expression of this gene.

To further test the reservoir model, we analyzed FUS expression under cellular stress conditions. Stress treatments have been reported to cause global mRNA decay (*35*, *36*). We found that FUSint6&7-RNA and its condensates become depleted during the recovery from arsenite stress, as well as in response to other chemical stresses (Fig. 4, D and E, and fig. S7, D and E). This was associated with maintained FUS mRNA level (Fig. 4, D and E), without significant changes in *FUS* transcription (pre-mRNA level; fig. S7F).

Last, we showed that FUS mRNA and protein were down-regulated following exoFUSint7 overexpression in FUSΔNLS neuroblastoma cells (Fig. 4, F and G). Of note, exoFUSint6/7 expression did not cause significant cellular toxicity (fig. S7G). Thus, the higher-order assemblies of FUSint6&7-RNA appear to be an integral part of normal FUS mRNA production, contributing to the regulation of this process in response to external cues.

### FUSint6&7-RNA condensates accumulate splicing factors and the m6A reader YTHDC1

**Fig. 5.**
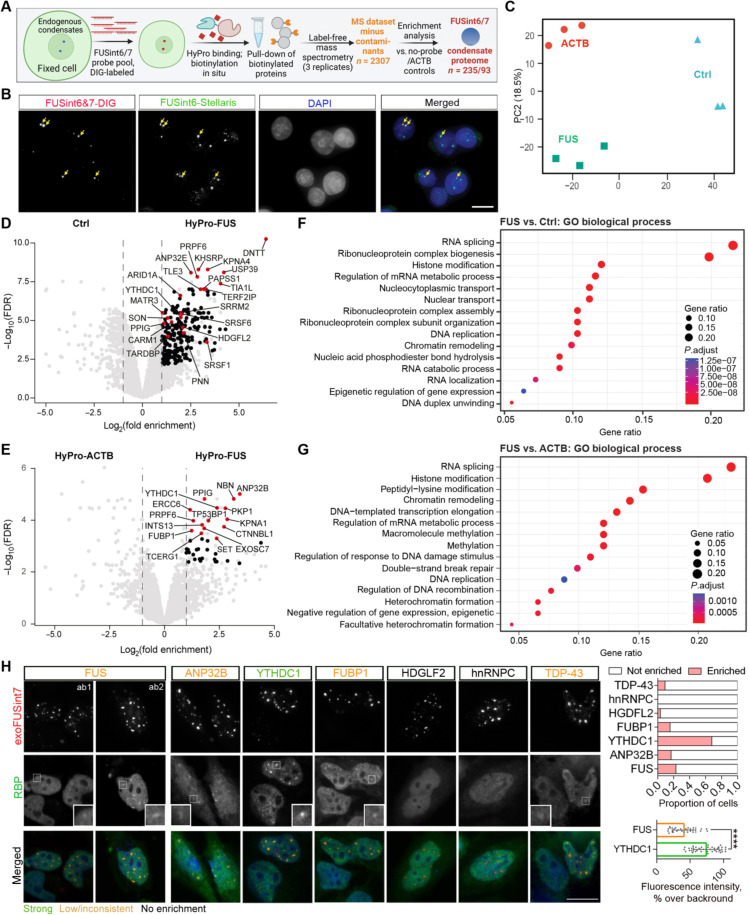
Proteomic analysis of FUSint6&7-RNA condensates by HyPro-MS. (**A**) Experimental pipeline for HyPro-MS analysis of FUSint6&7-RNA condensates. (**B**) Efficient labeling of the endogenous FUSint6&7-RNA condensates using HyPro probes. Stellaris FUSint6–specific probe was used for costaining. Arrows indicate the foci labeled by both Stellaris and HyPro probes. Scale bar, 10 μm. (**C**) Principal component analysis (PCA) demonstrating clustering of triplicated HyPro-MS samples for both probes and the no-probe control. (**D**) Volcano plot for FUSint6&7-RNA condensates versus no-probe control (Ctrl). Proteins with *P*adj < 0.05 are labeled in black, and proteins with *P*adj < 0.05 and involved in RNA splicing and/or implicated in neurodegeneration are labeled in red. (**E**) Volcano plot for FUSint6&7-RNA condensates versus ACTB probe. Proteins with *P*adj < 0.1 are labeled in black, and the top 15 hits are labeled in red. (**F**) Dot plot of Gene Ontology (GO) Biological Process term enrichment analysis for nuclear proteins identified in FUSint6&7-RNA condensates and significantly enriched as compared to no-probe control. (**G**) Dot plot of GO Biological Process term enrichment analysis for nuclear proteins identified in FUSint6&7-RNA condensates and significantly enriched as compared to ACTB probe control. (**H**) Validation of HyPro-MS proteins enriched in FUSint6&7-RNA condensates, as well as FUS and other proteins previously shown to bind FUSint6&7-RNA. Cells expressing exoFUSint7 were analyzed by RNA-FISH and immunofluorescence with appropriate antibodies. ab1,2 - different FUS antibodies. Representative images are shown. Scale bar, 15 μm. Top graph: Twenty-eight to 35 transfected cells with condensates were analyzed per protein. Bottom graph: Forty-one and 27 individual condensates were analyzed for YTHDC1 and FUS enrichment, respectively, ****P < 0.0001, two-tailed unpaired *t* test.

To gain insights into FUSint6&7-RNA condensate composition and hence regulation, we performed hybridization-proximity labeling coupled with mass spectrometry (HyPro-MS) (Fig. 5A) (*37*, *38*). We previously used this approach to analyzing protein and RNA interactomes of RNA-seeded compartments in genetically unperturbed cells. Here, we leveraged a modified (HyPro2) version of the procedure, which allows efficient labeling of small RNP structures while reducing the unspecific diffusion of activated biotin. A probe set covering FUS introns 6 and 7 (68 oligonucleotides in total) was used, alongside two controls: a no-probe control (Ctrl) and a β-actin (ACTB) intronic probe set (34 oligonucleotides). The latter probe detected small foci in HeLa cells corresponding to newly produced pre-mRNAs (fig. S8A)—and therefore was ideally suited as a control for the analysis of FUSint6&7-RNA–specific interactors. Efficient labeling of FUSint6&7-RNA condensates in a HyPro experiment was confirmed by combining HyPro-FISH with FUSint6&7-RNA–specific probes and RNA-FISH with the FUS intron 6 Stellaris probe set (Fig. 5B).

Following the hybridization with digoxigenin (DIG)-labeled probes and incubation with the HyPro enzyme (see Materials and Methods for details), the molecules physically proximal to the FUSint6&7-RNA transcripts (bait) were biotinylated in situ and captured on streptavidin beads under denaturing conditions. The expected enrichment of biotinylated RNA “baits” on the beads was confirmed by qRT-PCR (fig. S8B). Efficient protein biotinylation in all samples was confirmed by Western blotting (fig. S8C). Label-free mass spectrometry of the captured proteins identified a total of 2869 proteins—reduced to 2307 after additional filtering steps [removal of common contaminants, including those from the CRAPome database (*39*)]. Principal component analysis (PCA) of the filtered proteins demonstrated good clustering of the triplicates for each condition (Fig. 5C).

Enrichment analysis for FUSint6&7-RNA condensates as compared to the no-probe control identified 235 proteins (*P* < 0.05), of which 184 were significant after correction for multiple comparisons (*P*adj < 0.05) (Fig. 5D and table S2). Only 93 proteins were significantly enriched when compared to ACTB probe (*P* < 0.05), of which 19 remained significant after correction for multiple comparisons (*P*adj<0.05) (Fig. 5E and table S2). FUSint6&7-RNA condensates were enriched in factors involved in RNA splicing, histone modification, and RNP complex assembly (Fig. 5F). When compared to ACTB intronic foci, they also showed enrichment for the factors related to macromolecule methylation and DNA damage repair (Fig. 5G). The top 10 hits (versus ACTB probe) included nuclear speckle components such as SR proteins and PPIG, as well as the nuclear m6A reader YTHDC1. These condensates were also found to recruit ALS-linked proteins TDP-43, MATR3, EWS, and hnRNPA3 (Fig. 5, D and E, and table S2).

Given the importance of FUS intron 7 for condensate assembly established in the above experiments (Fig. 3), we used exoFUSint7 for immunofluorescence validation of the HyPro-MS hits. In addition to the top hits ANP32B, YTHDC1, FUBP1, PPIG, and TCERG1, we included proteins with known binding motifs in FUS introns 6/7 [RBPDB database (*40*)] such as HDGFL2 and hnRNPC, as well as TDP-43 and FUS itself. YTHDC1 was found to be highly enriched in >60% of all exoFUSint7 condensates, and TDP-43, ANP32B, and FUBP1 also showed some enrichment in a fraction (<20%) of condensates (Fig. 5H). Other protein candidates showed no detectable enrichment (Fig. 5H), indicating that they may interact with the sequences outside of intron 7 or associate with the condensates only transiently. FUS protein, which showed only minimal enrichment in the HyPro-MS analysis using ACTB as a control [1.05-fold log fold change (FC) versus ACTB, *P* = 0.044, *P*adj = 0.23], displayed some recruitment into ~20% of exoFUSint7 condensates—albeit significantly weaker compared to YTHDC1 (Fig. 5H). Thus, FUSint6&7-RNA condensates interact with a distinct set of proteins involved in posttranscriptional regulation of gene expression.

### m6A and YTHDC1 promote FUSint6&7-RNA condensation

**Fig. 6.**
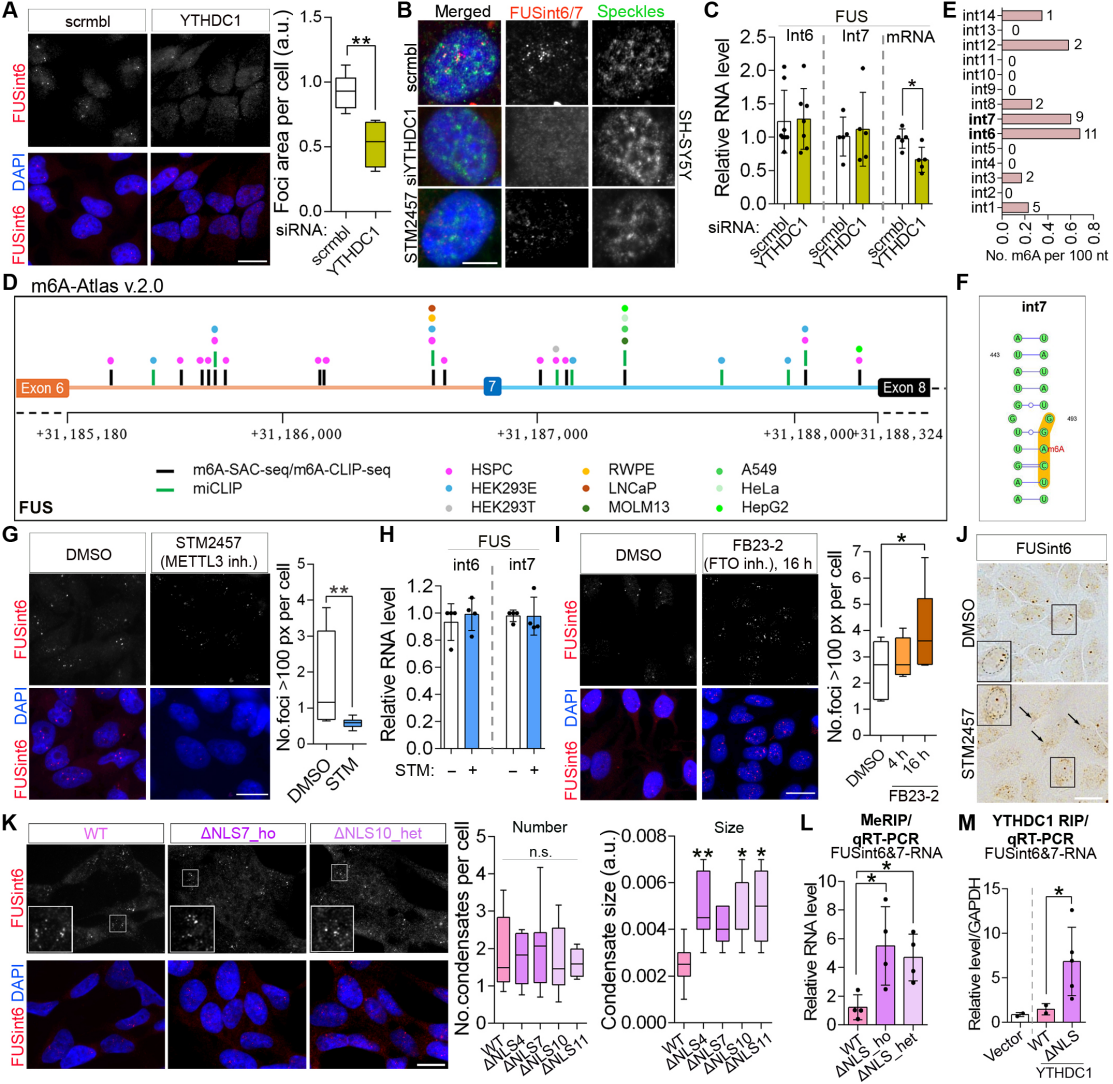
m6A/YTHDC1 maintain FUSint6&7-RNA condensates. (**A** and **B**) YTHDC1 depletion destabilizes FUSint6&7-RNA condensates. Representative images and quantification of condensates (A) and high-resolution images (B) are shown. One hundred seven and 65 cells (four to five FOVs) were analyzed for scrambled (scrmbl) and YTHDC1 siRNA, respectively. **P < 0.01, Mann-Whitney *U* test. (**C**) YTHDC1 depletion does not affect the FUSint6&7-RNA level but down-regulates FUS mRNA. *N* = 5 to 7, *P < 0.05, Mann-Whitney *U* test. (**D** and **E**) FUS introns 6 and 7 are extensively methylated. m6A-Atlas 2.0 was used for mapping methylation sites (D) and calculating the m6A mark density (E). Colored dots indicate cell lines. (**F**) A high-confidence DRACH motif in FUS intron 7 shown in a structural context. (**G** and **H**) Pharmacological inhibition of a m6A writer METTL3 destabilizes FUSint6&7-RNA condensates (G) without changing levels of this RNA (H). In (G), cells were treated for 16 hours, and >200 cells (five FOVs) were analyzed per condition, **P < 0.01, Mann-Whitney *U* test. In (H) (qRT-PCR), *N* = 4. (**I**) Pharmacological inhibition of a m6A eraser FTO promotes FUSint6&7-RNA condensate assembly. Seventy-nine, 106, and 117 cells (five FOVs) were analyzed for dimethyl sulfoxide (DMSO), 4- and 16-hour FB23-2 treatments, respectively. *P < 0.05, Kruskal-Wallis with Dunn’s test. (**J**) FUSint6 RNAscope-ISH reveals partial cytoplasmic redistribution of FUSint6&7-RNA in STM2457-treated cells. Arrows indicate cytoplasmic accumulations of this RNA; nucleus is circled. (**K**) Preserved or enhanced formation of FUSint6&7-RNA condensates in FUSΔNLS lines. Two hundred thirty-four, 239, 234, 141, and 62 cells were analyzed for WT, ΔNLS4, ΔNLS7, ΔNLS10, and ΔNLS11 lines, respectively (four to nine FOVs). *P < 0.05 and **P < 0.01, Kruskal-Wallis with Dunn’s test. (**L**) m6A RNA immunoprecipitation (MeRIP) coupled with qRT-PCR demonstrates increased FUSint6&7-RNA methylation in FUSΔNLS lines. *N* = 4, *P < 0.05, Kruskal-Wallis with Dunn’s test. (**M**) Enhanced association of FUSint6&7-RNA with YTHDC1 in FUSΔNLS lines, as demonstrated by RNA immunoprecipitation (RIP). Data were combined for three ΔNLS lines. *P < 0.05, Mann-Whitney *U* test (WT versus ΔNLS). Scale bars, 5 μm in (B) and 20 μm in other panels.

The m6A reader YTHDC1 was strongly enriched in FUS RNA condensates according to HyPro-MS (in the top five enriched proteins versus ACTB probe; FC = 2.41, *P*adj = 0.011) and immunofluorescence validation (Fig. 5H). YTHDC1 knockdown destabilized these condensates (Fig. 6, A and B, and fig. S9A), without altering the total FUSint6&7-RNA level (Fig. 6C). FUS mRNA was down-regulated following YTHDC1 knockdown (Fig. 6C), in line with the contribution of condensation to posttranscriptional splicing of FUSint6&7-RNA.

YTHDC1 enrichment suggested that either FUSint6&7-RNA itself or other RNAs in these condensates are methylated. Analysis of public datasets via the m6A-Atlas 2.0 aggregator (*41*) confirmed multiple m6A marks in both FUS introns 6 and 7 in most of the cell lines (11 and 9 in introns 6 and 7, respectively) (Fig. 6D and table S3). When normalized to intron length, these two introns were found to harbor more m6A marks than other *FUS* introns (Fig. 6E and table S3). Analysis of m6A modification consensus motifs (DRACH) revealed that FUS intron 7 contains three canonical GGACU/A motifs, which are most prevalently methylated in human cells (*42*). For comparison, only one such motif is present in introns 1 and 8 each and none in other FUS introns (table S3). At least one of these motifs in FUS intron 7 is a predicted high-confidence m6A site when taking into the account the structural context (Fig. 6F) (*43*). Last, FUS intron 7 also contains a YTHDC1 binding motif GAAUGC (*40*).

To address whether methylation levels directly contribute to FUSint6&7-RNA condensate integrity, we used STM2457, a specific inhibitor of a major m6A writer/methyltransferase, METTL3 (*44*). Similar to YTHDC1 knockdown, pharmacological depletion of the m6A mark destabilized condensates without affecting the FUSint6&7-RNA level (Fig. 6, B, G, and H). This result was corroborated by small interfering RNA (siRNA)–mediated depletion of another m6A writer, METTL16 (fig. S9B). Conversely, m6A up-regulation by FB23-2, a small-molecule inhibitor of an m6A eraser FTO (*45*), increased the condensate abundance (Fig. 6I). FUSint6 RNAScope-ISH also revealed cytoplasmic redistribution of FUSint6&7-RNA in STM2457-treated cells (Fig. 6J), indicating that loss of condensate integrity is associated with reduced nuclear retention.

Unexpectedly, the numbers of FUSint6&7-RNA condensates remained unchanged, and their size increased in FUSΔNLS neuroblastoma cell lines as compared to WT cells (Fig. 6K), despite FUSint6&7-RNA down-regulation (Fig. 1; heated samples were used for qRT-PCR analysis, ruling out the contribution of RNA semiextractability). We hypothesized that this effect could be due to increased condensation of FUSint6&7-RNA in response to elevated m6A methylation. m6A RNA immunoprecipitation coupled with qRT-PCR revealed a significant gain in m6A modification on FUSint6&7-RNA in these mutant cell lines (adjusted for the total transcript level; Fig. 6L). To corroborate this result, we performed RNA immunoprecipitation in WT and FUSΔNLS cells using transient expression of YTHDC1-Flag and measured FUSint6&7-RNA levels by qRT-PCR. MALAT1, which is extensively m6A-modified (*46*), demonstrated ~40-fold enrichment in the YTHDC1-Flag immunoprecipitation (IP) samples as compared to the vector-only control, confirming successful pull-down (fig. S9C). FUSint6&7-RNA was markedly enriched in YTHDC1-Flag IP preparations from FUSΔNLS cells compared to WT cells (Fig. 6M). Of note, some down-regulation of YTHDC1 mRNA was detectable by RNA sequencing in the heterozygous FUSΔNLS lines (fig. S9D); however, the abundance and distribution of YTHDC1 protein remained unchanged (fig. S9E). Global m6A levels were also unaltered in these cell lines, as indicated by dot blot and enzyme-linked immunosorbent assay (ELISA) analyses (fig. S9, F and G). We concluded that mutant FUS drives an increase in m6A modification of FUSint6&7-RNA, which in turn promotes condensation of this RNA in the nucleus.

### m6A modification limits FUS protein association with RNA

**Fig. 7.**
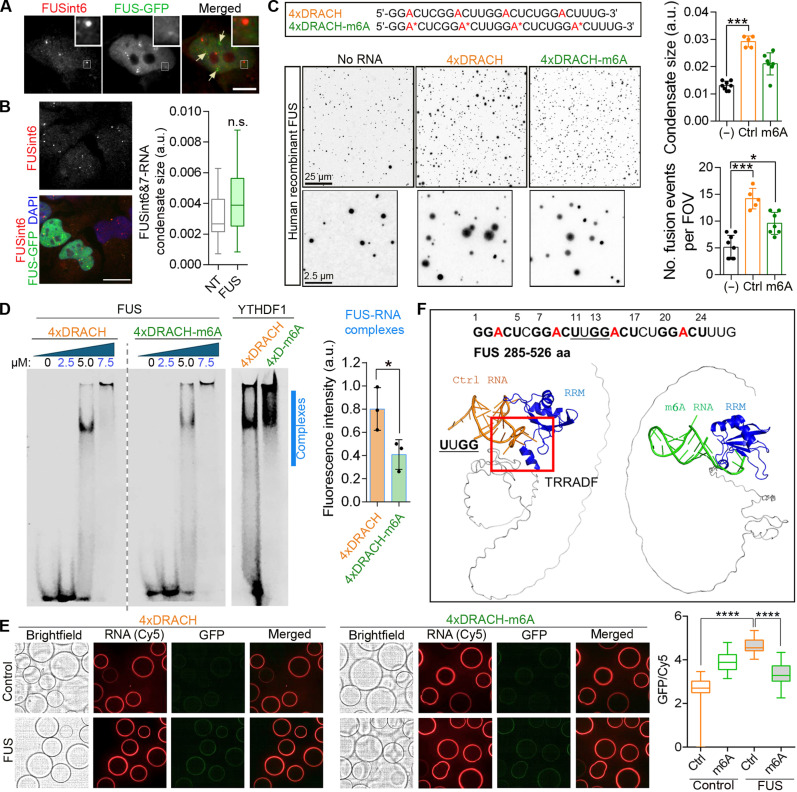
m6A has a repelling effect on FUS protein. (**A**) Ectopically expressed GFP-tagged FUS is not enriched in the endogenous FUSint6&7-RNA condensates. FUSint6 Stellaris probe was used in HeLa cells. Note FUS protein accumulation in paraspeckles (arrows). Scale bar, 5 μm. (**B**) FUS overexpression does not affect the FUSint6&7-RNA condensate size. NT, nontransfected (cells analyzed in the same FOV). Condensate size was measured in 15 FOVs per condition. Scale bar, 10 μm. (**C**) M6A limits FUS condensation in vitro. Recombinant human his-tagged FUS and synthetic Cy5-labeled RNA oligonucleotides, fully methylated or unmodified, were used. Representative images of FUS condensates and quantification of average condensate size and fusion events are shown. Five to eight FOVs were analyzed per condition from a representative experiment. *P < 0.05 and ***P < 0.001, Kruskal-Wallis with Dunn’s test. (**D**) FUS-RNA complex formation is diminished by methylation, as analyzed by electrophoretic mobility shift assay. Recombinant FUS protein and RNA oligonucleotides as in (C) were used. Recombinant YTHDF1 protein was used in parallel as a positive control. Complex formation in the indicated area (blue line) was quantified by densitometry (5.0 and 7.5 μM data points combined). *N* = 3, *P < 0.05, Mann-Whitney *U* test. (**E**) Confocal nanoscanning (CONA) assay demonstrates reduced binding of FUS to methylated RNA, as compared to its unmodified counterpart. Cy5-labeled RNA oligonucleotides as above and lysates of cells expressing GFP-tagged FUS were used. GFP ring fluorescence intensity was normalized to Cy5 fluorescence–RNA coating. Eighty to 100 beads were analyzed per condition. ****P < 0.0001, Kruskal-Wallis with Dunn’s test. (**F**) AlphaFold3 modeling confirms the repelling properties of m6A in FUS binding to RNA. FUS RRM is in blue, and RNA is in orange (unmodified) or green (methylated). Interacting interfaces of FUS protein and the DRACH motif–containing RNA are boxed. Amino acid (aa) sequence interacting with the UUGG motif in the oligonucleotide is also indicated.

FUS protein has multiple binding sites in FUS introns 6 and 7 according to iCLIP (*19*, *47*), yet we observed only modest FUS protein recruitment into FUSint6&7-RNA condensates in HyPro-MS and immunostaining validation studies (Fig. 5). Ectopically expressed FUS-GFP also did not display detectable enrichment in endogenous FUSint6&7-RNA condensates and had no effect on the condensate size (Fig. 7, A and B). Since m6A can both attract and repel RBPs (*48*), we hypothesized that this modification on FUSint6&7-RNA may regulate FUS protein association with these condensates.

We first used the in vitro ImmuCon assay for condensate reconstitution in vitro that we recently developed (*49*) to test the effect on m6A on FUS interaction and condensation with RNA. Although FUS binds RNA with limited sequence specificity, it has some preference for pentamers containing GG upstream of uridines (*47*). We used an RNA oligonucleotide containing four repeats of the natural DRACH motif GGACU present in FUS intron 7 (“4xDRACH”) and its fully modified counterpart (“4xDRACH-m6A”) (Fig. 7C). Recombinant FUS protein was mixed with RNA at a final concentration of 2.5 μM [previously found optimal for condensate formation (*49*)], sedimented on glass coverslips, fixed, and analyzed by immunostaining. 4xDRACH RNA promoted FUS condensate formation, leading to large, frequently fusing condensates—suggestive of their high fluidity (Fig. 7C). Although 4xDRACH-m6A RNA did increase FUS condensation compared to the “no-RNA” control, the resultant condensates were significantly smaller and fused less as compared to the nonmethylated RNA (Fig. 7C). M6A was previously reported to enhance phase-separation properties of RNA (*50*). 4xDRACH-m6A underwent more efficient condensation than its nonmodified counterpart (fig. S10A), suggesting that its homotypic condensation may have outcompeted co-condensation with FUS. Electrophoretic mobility shift assay also revealed more efficient formation of FUS-RNA complexes with 4xDRACH RNA as compared 4xDRACH-m6A RNA (Fig. 7D).

To further corroborate these findings, we used a version of the on-bead confocal nanoscanning (CONA) assay (*51*), where ring formation by a GFP-tagged RBP from cell lysate on RNA-coated beads can be used as a measure of its LLPS (*52*). We first confirmed that a positive control—m6A reader YTHDF1—accumulated on the beads coated with either unmodified or m6A-modified 4xDRACH RNA, with a clear preference for the latter (fig. S10B). Analysis of FUS protein samples demonstrated diminished ring signal intensity on the beads coated with 4xDRACH-m6A RNA as compared to unmodified RNA (Fig. 7E).

Last, we used AlphaFold3 to predict interactions between FUS RNA binding sequences (C terminus, amino acids 285 to 526) and the above RNA oligonucleotides. This analysis revealed an interaction between a sequence in the FUS RRM (amino acid TRRADF) and the middle portion of 4xDRACH RNA (UUGG) but not its methylated counterpart (Fig. 7F). These data suggest that FUS protein is excluded during condensation of m6A-modified RNA, reducing its enrichment in FUSint6&7-RNA condensates, possibly due to a direct repulsion by the m6A mark.

## DISCUSSION

**Fig. 8.**
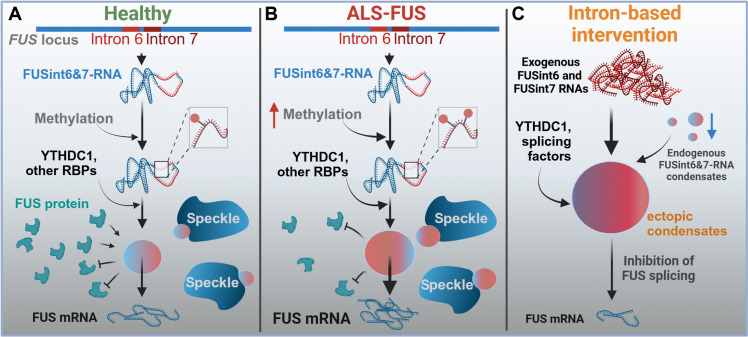
Regulation of FUS RNA condensation and posttranscriptional splicing in WT and ALS-FUS cells and the effect on exogenous FUS introns. (**A**) Regulation of FUS expression by RNA condensation in healthy cells. (**B**) Dysregulated FUS RNA condensation in ALS-FUS cells. (**C**) Approach to FUS expression regulation based on the intron-mediated condensation mechanism.

Our work establishes that RNA condensation and delayed posttranscriptional splicing play an important role in FUS expression. This regulation relies on a relatively abundant and stable FUS splicing intermediate that retains two conserved introns, 6 and 7. First, we demonstrate that this RNA species can assemble into higher-order RNP structures that contribute to FUS mRNA production. Second, our data suggest that gain-of-function mutations in FUS trigger a switch from a negative- to a positive-feedback autoregulation mode, with major implications for ALS-FUS pathology. Last, we provide evidence that the retained introns themselves can be exploited to control FUS expression in the disease context (Fig. 8).

### Relationship between retained introns, RNA condensates, and FUS expression

We show that FUS transcripts retaining introns 6 and 7 form nuclear condensates in cultured cells including neurons. In particular, FUS intron 7 in isolation is prone to forming microscopically visible foci and may thus drive the condensation behavior of the entire transcript. RNAfold predictions indicate that intron 7 is more “structured” than the nonfoci-forming intron 6, featuring higher base-pairing probabilities and lower ensemble diversity. Highly structured RNAs were previously shown to form globular condensates, in contrast to “disordered” RNAs that tend to form mesh-like networks (*53*). However, we also found that none of the three parts of intron 7, when expressed in isolation, can fully reproduce the condensation behavior. It is possible that long-range interactions between portions of intron 7 are important for condensation. Nevertheless, the most structured part of intron 7 had the highest propensity to form large condensates.

The characteristic number of large FUSint6&7-RNA foci per nucleus points to their assembly near the *FUS* transcription sites (Fig. 2). Yet, several lines of evidence suggest that these structures are distinct from the clusters of pre-mRNA molecules occasionally observed near transcription sites of actively expressed genes (e.g., *ACTB*; fig. S8). First, these FUSint6&7-RNA foci are substantially larger than pre-mRNA clusters detected at the *ACTB* transcription sites (compare Fig. 2 and fig. S8), despite the steady-state expression of *ACTB* is ~18-fold higher than that of *FUS* [6761 nTPM (transcripts per million) versus 376 nTPM, respectively; Human Protein Atlas]. Second, smaller FUSint6&7-RNA condensates are often detected in addition to the main foci—also associated with speckles (Figs. 2 and 3), whereas no such “secondary” condensates form in the case of *ACTB*. Third, FUSint6&7-RNA appears to be abundant than fully unspliced FUS pre-mRNA species (fig. S3), being sufficiently stable to act as a structural component of RNP condensates. The behavior of FUSint6&7-RNA is reminiscent of that of NEAT1_2, a structural scaffold for paraspeckles. Paraspeckles cluster at the site of transcription, with a fraction of individual particles “budding” off this site and attaching to (but not fusing with) nuclear speckles (*29*).

The proximity of FUSint6&7-RNA condensates to nuclear speckles (Fig. 3 and fig. S4) and the abundance of splicing factors in their interactome/proteome (Fig. 5) support a model in which these transcripts act as “reservoirs” of relatively long-lived splicing intermediates that can rapidly yield mature FUS mRNA, in a condition-dependent manner. Notably, posttranscriptional splicing accounts for ~20% of splicing in human cells, often occurring near nuclear speckles (*54*, *55*). Dispersal of speckles by knocking down their core protein SON increases retention of speckle-associated introns (*56*). Thus, nuclear speckle proximity of the FUSint6&7-RNA condensates might facilitate the final steps of FUS mRNA biogenesis. Although further work will be needed to assess the dynamic range of this “reservoir” mechanism, we propose that it may buffer gene expression changes under stress conditions. A recent study using direct, full-length RNA sequencing in arsenite-treated cells has demonstrated that stress induces transcriptome-wide mRNA decay (*36*). Enhanced FUSint6&7-RNA splicing might therefore be important to maintain the FUS mRNA level for optimal recovery from stress.

Stimulus-dependent splicing of retained introns has been previously documented, including in neuronal contexts (*57*–*59*). Extending the scope of such regulation, a group of retained introns in mammals, termed “detained introns,” has been identified on the basis of their ability to accumulate in the nucleus before undergoing posttranscriptional splicing (*60*). The high interspecies conservation of FUS introns 6 and 7, their responsiveness to external stimuli, relative stability, nuclear retention, and NMD resistance indicate that these introns belong to the above category. In line with detained introns responding to DNA damage (*60*), HyPro-MS highlighted the presence of DNA damage repair factors in FUSint6&7-RNA condensates.

We previously described the formation of phase-separated structures in the nucleus for Srsf7 RNA with retained introns (*27*). Future studies should explore the extent to which condensation of stable RNA species with retained introns contributes to posttranscriptional splicing regulation and the dynamics of nuclear RNA reservoirs. Functional interaction between such condensates and other membraneless organelles in the nucleus would be another avenue of future investigation (*61*).

### ALS-FUS mutations rewire FUS RNA condensate regulation

An important finding of our study is the role of the m6A reader YTHDC1 in the FUSint6&7-RNA condensate regulation (Fig. 6). m6A modification is emerging as a key factor in RNA compartmentalization and fate in the nucleus. For example, m6A modification of MALAT1 significantly modulates nuclear speckle composition and properties (*46*), whereas enhancer RNA methylation facilitates enhancer RNA condensate assembly (*62*). The m6A mark has a direct condensation-promoting effect in vitro (*50*). In cells, YTHDC1, which has two intrinsically disordered regions and is capable of phase separation, likely also contributes to RNA condensation (*63*). Furthermore, RNA condensation via m6A/YTHDC1 was reported to promote nuclear retention of RNA (*64*) and protect RNA from degradation (*65*). Our study expands the repertoire of m6A/YTHDC1-dependent nuclear condensates.

Previous studies proposed two negative-feedback mechanisms maintaining FUS homeostasis (*18*, *19*). These mechanisms rely on FUS protein binding to the intron 6–exon 7–intron 7 region of its pre-mRNA, promoting either exon skipping or intron retention. While loss of WT FUS in our KO cell model did not alter FUSint6&7-RNA abundance (Fig. 1), our intron 7 overexpression studies suggested that some FUS recruitment onto its own transcript takes place (Fig. 5), consistent with the published iCLIP data (*47*). Unlike FUS KO, ALS-FUS mutations had a suppressive effect on FUSint6&7-RNA in our studies (Fig. 1). This aligns with reduced intron 6/7 retention observed in three ALS-FUS mouse models but not in three FUS KO models in a previous study [figure 4C in (*19*)]. Our data on m6A modification may explain this behavior. FUS RNA methylation levels and YTHDC1 recruitment significantly increase in ALS-FUS mutant cells (Fig. 6). This in turn potentiates the assembly of FUSint6&7-RNA condensates—positive regulators of FUS posttranscriptional splicing (Fig. 6). Simultaneously, m6A-mediated condensation may hinder FUS protein access to its binding sites within the intron 6–exon 7–intron 7 region (Fig. 7). We propose that ALS-FUS mutations rewire the posttranscriptional regulation of FUS, disrupting its self-inhibitory feedback loop and, at the same time, inducing abnormal self-activation (Fig. 8, A and B). FUS accumulation is typical for both ALS-FUS models and patient tissue (*14*, *16*, *17*), and even a modest increase in the FUS level is toxic in vivo (*66*, *67*). The mutation-induced switch in the FUS regulatory logic we describe will have profound implications for its expression control and ALS pathology.

An important question for future studies is why ALS-FUS mutations increase m6A modification of FUS RNA. One possibility is that the mutation-induced shift of FUS protein to the cytoplasm leaves its RNA more exposed to m6A writers. Alternatively, this effect may arise through indirect mechanisms, including disruption of RBP regulatory networks (*68*–*70*). Indeed, mutant FUS has been shown to reduce the solubility of multiple RBPs (*9*).

### Therapeutic potential of FUS introns

A recent study by the Dupuis group (*20*) has described a notable amelioration of mutant FUS toxicity in mice upon introduction of a full human *FUS* transgene, including the rescue of lethality/shortened life span and paralysis in homozygous and heterozygous Fus^∆NLS^ mice, respectively. This was associated with accumulation of endogenous FUSint6&7-RNA and reduced (mutant) FUS mRNA and protein production. Phenotype rescue in these double-transgenic mice seemed counterintuitive, since FUS overexpression was toxic in other FUS transgenic mice (*66*, *67*). The key difference in the study design was the presence of all *FUS* regulatory sequences in the transgene used in this recent study. Our data (Fig. 4) argue that FUS intron 7 may be responsible for the therapeutic effect of the full *FUS* transgene. Exogenous expression of FUS intron 7—alone or in combination with intron 6—leads to ectopic condensate formation and sequestration of certain splicing factors, YTHDC1, and, potentially, FUSint6&7-RNA itself (Fig. 8C). Depletion of these factors results in the dissolution of endogenous condensates, inhibited posttranscriptional splicing, and, ultimately, FUS mRNA and protein down-regulation (Fig. 8C). Coincidentally, FUS ASO Jacifusen targets FUS intron 6 (*9*)—where both FUS pre-mRNA and FUSint6&7-RNA are expected to be degraded. Thus, FUSint6&7-RNA (condensate) depletion may contribute to efficient FUS mRNA down-regulation by this ASO. Notably, in our cellular studies, even high levels of exoFUSint6/7-RNA were not cytotoxic. It is possible that these ectopic condensates sequester a limited subset of splicing factors, causing no major changes to the global splicing patterns. In the future studies, it would be important to verify and fully characterize the rescue effect of FUS introns in human neuronal models (e.g., using viral vectors), before considering translational development of this approach. In addition, FUS introns are relatively long, and minimal a regulatory sequence(s) may have to be established. In conclusion, our study pinpoints a regulatory element in the *FUS* gene that may confer a therapeutic effect in ALS.

## MATERIALS AND METHODS

### Bioinformatic analyses

The human genome sequence (GRCh38.primary_assembly.genome.fa) and transcriptome annotation (gencode.v32.basic.annotation.gtf) were obtained from GENCODE (https://gencodegenes.org/human/release_32.html). Retention patterns of FUS introns 6 and 7 were analyzed using Illumina sequencing data for clone S3 of HeLa cells (*21*) (https://encodeproject.org/; Thomas Gingeras lab; https://ncbi.nlm.nih.gov/sra runs SRR4235529 and SRR4235530) and control human motor neurons (*22*) (https://ncbi.nlm.nih.gov/sra runs SRR8083865, SRR8083866, SRR8083872, SRR8083873, SRR8083878, and SRR8083879). RNA sequencing data were aligned to the GRCh38/hg38 primary assembly genome using HISAT2 (*71*). The aligned data were converted from SAM to sorted and indexed BAM format using SAMtools (*72*) and visualized in IGV (https://igv.org/). For simplicity, the minimum exon junction coverage in IGV sashimi plots was set to 5% of the expected junction coverage, which was estimated as the total number of *FUS*-specific junction reads divided by 14—the number of introns in the representative FUS transcript ENST00000254108.

To analyze the mutual retention status of introns 6 and 7, aligned reads spanning exon 7 with ≥2-nucleotide overhangs on both sides were selected using BEDTools (*73*). These reads were then classified in R (https://r-project.org/) into one of the four possible patterns: Both introns 6 and 7 spliced; intron 6 retained and intron 7 spliced; intron 6 spliced and intron 7 retained; or both introns 6 and 7 retained. Retention efficiencies for each intron, derived from this analysis, were used to estimate isoform distribution under the assumption of independent excision of introns 6 and 7, using a Punnett square-like approach.

Comparisons of *FUS* introns 6 and 7 to all internal introns (i.e., nonfirst and nonlast) were performed in R, using transcripts from the gencode.v32.basic.annotation.gtf file that contained at least three introns. Intron length was calculated on the basis of genomic coordinates, and GC content was obtained using the bioseq package (https://cran.r-project.org/web/packages/bioseq/). MaxEnt scores for splice donors (5′ss) and acceptors (3′ss) were computed by running the score5.pl and score3.pl Perl scripts locally (*23*). Distances from the branch point to the 3′ss were retrieved from table S2 of (*25*) and mapped to GRCh38/hg38 coordinates using the UCSC LiftOver tool (https://genome.ucsc.edu/cgi-bin/hgLiftOver). Donor and acceptor sites of introns 6 and 7 were also compared to other *FUS* introns (including the first and the last ones) using the appropriate online MaxEntScan tools (hosted at https://genes.mit.edu/).

For FUS introns 6 and 7 structure prediction and visualization, RNAfold v2.0 (*34*) was used, with the parameters described earlier (*74*). FUS m6A methylation data were obtained from m6A-Atlas 2.0 (http://rnamd.org/m6a/) (*41*) and viewed in the UCSC Genome Browser. Predictions of FUS interaction with unmodified and m6A-modified 4xDRACH oligonucleotides were performed using AlphaFold3, visualizing the top-ranked results in PyMOL v.2.55.5.

### Expression constructs

Plasmids for ectopic expression of FUS introns were generated by amplifying the intron sequences with a portion of the flanking exon sequences, preserving the splice site; primers are given in table S4. Resultant fragments were inserted in pEGFP-N1 vector between Xho I and Kpn I sites (upstream of GFP open reading frame). Plasmids for the expression of full-length FUS intron 7 without splice sites and the three segments/regions were custom made by Genewiz, in the same vector. Plasmids for GFP-tagged WT and mutant FUS expression were described previously (*75*).

### Cell culture experiments

SH-SY5Y, HeLa, and U2OS WT cells from the European Collection of Authenticated Cell Cultures (ECACC) repository were maintained in Dulbecco’s modified Eagle’s medium/F12 media supplemented with 10% foetal bovine serum and 1× penicillin-streptomycin-glutamine (all from Invitrogen). FUSΔNLS lines were generated and characterized previously (*17*). Cells were plated on uncoated 10-mm coverslips in 24-well plates and left to recover overnight before transfection or treatments. Cells were transfected with plasmid DNA using either Lipofectamine 2000 (Invitrogen) or JetPRIME reagent (Sartorius), according to the manufacturer’s instructions. siRNA transfections were done using Lipofectamine 2000. Silencer Select–validated siRNAs for YTHDC1 and METTL16 were purchased from Thermo Fisher Scientific. AllStars scrambled siRNA (QIAGEN) was used as a control. The following chemical modulators of cellular pathways were used (final concentration): sodium arsenite (0.5 mM), cycloheximide (10 μM), actinomycin D (2.5 μg/ml), IFN-β (1 x 10^4^ IU) (all from Sigma-Aldrich); pladienolide B (200 nM), STM2457 (10 μM), and FB23-2 (10 μM) (all from Cayman Chemicals). Cell survival analysis was performed 24 hours posttransfection using CellTiter-Blue Cell Viability Assay (Promega) according to the manufacturer’s instructions, in a 96-well format, after verification of transfection efficiency. For 1,6-HD, DNase I, and RNase A treatments in semipermeabilized cells, HeLa cells were treated with 1% Tween/phosphate-buffered saline (PBS) for 10 min before subjecting to a treatment: 5% 1,6-HD in PBS for 5 min, DNase I (0.2 mg/ml), or RNase A (QIAGEN) diluted in PBS for 20 min, followed by fixation as normal. Human motor neurons were differentiated from human embryonic stem (ES) cells (H9 line) and maintained as described in (*76*).

### Generation of cell clones with FUS intron 6 partial deletion

For generation of cell clones with FUS intron deletion (Δint6), sgRNA sequences were selected using the Integrated DNA Technologies (IDT) guide RNA online tool: forward, AACCATTACCAACTCCCTAG; reverse, TGTCAACCACTTGCGACAAG. Annealed DNA oligonucleotides (custom-made by Merck) were cloned into a pX330 vector (*77*) (Addgene plasmid #42230). HeLa cells were transfected with both plasmids using JetPRIME, subjected to limited dilution, and single-cell–derived clones were analyzed by PCR (primers are given in table S4) using Go-Taq Green Master Mix (Promega). Successful editing was confirmed by DNA sequencing.

### RNA-FISH and immunocytochemistry

Cell fixation, processing, RNA-FISH, and immunocytochemistry on coverslips were performed as described earlier (*17*). Fluorescently labeled RNA-FISH probes for FUS introns 1, 6, and 7 were designed and custom-made by Biosearch Technologies (Stellaris probes). Oligonucleotide sequences for probe pools are provided in table S5. FUS intron 6 and 7 probes were labeled with Quasar570, and intron 1 probe was labeled with fluorescein isothiocyanate (FITC). The single DNA oligonucleotide probe used for exoFUSint7 condensate detection was 5′end labeled with Cy5: 5′-TCCCGAGGGCCTTTAGTGAC-3′ (custom-made by Merck). Paraspeckles were detected using a NEAT1_2-specific Stellaris custom probe labeled with Quasar670, and speckles were labeled with a custom Cy5-labeled poly(dT)_30_ probe (Sigma-Aldrich). RNA-FISH in a 96-well format was carried out as described before (*78*), except the final probe concentration was 50 nM. For immunocytochemistry, cells prepared as for RNA-FISH were washed with 1× PBS and incubated in a primary antibody (1:1000) for 2 hours and subsequently in a secondary antibody (1:1000; Alexa Fluor, Invitrogen) for 1 hour at room temperature (RT). The following commercial primary antibodies were used: FUS (mouse monoclonal, Santa Cruz, sc-47711, RRID:AB_2105208 and rabbit polyclonal, Proteintech, 11570-1-AP, RRID:AB_2247082); SMN (mouse monoclonal, BD Biosciences, 610646, RRID:AB_397973); coilin p80 (mouse monoclonal, BD Biosciences, 612074, RRID:AB_2081554); TDP-43 (rabbit polyclonal, C-terminal, Sigma-Aldrich, T1580, AB_2532125); YTHDC1 (rabbit polyclonal, Proteintech, 14392-1-AP, RRID:AB_2878052); HDGFL2 (rabbit polyclonal, Proteintech, 15134-1-AP, RRID:AB_2117220); hnRNPC (rabbit polyclonal, Proteintech, 11760-1-AP, RRID:AB_2117500); PNN/pinin (rabbit polyclonal, Proteintech, 18266-1-AP, RRID:AB_10642138); ANP32B (rabbit polyclonal, Proteintech, 10843-1-AP, RRID:AB_2056460); and FUBP1 (rabbit polyclonal, Proteintech, 24864-1-AP, RRID:AB_2879762).

### Fluorescent cell imaging and analysis

For imaging on coverslips, an Olympus BX57 fluorescent microscope (100× oil objective) equipped with an ORCA-Flash 4.0 camera (Hamamatsu) and cellSens Dimension software (Olympus) was used. Super-resolution imaging of condensates was performed on Zeiss Airyscan 2, and images were processed using the Zeiss ZEN blue software. Imaging on Opera Phenix was performed at 80% laser power and 500-ms exposure for the Cy3 channel. FUS RNA condensate properties (area, number, solidity, and fluorescence intensity) were quantified using the Analyze particles tool in ImageJ (https://imagej.net/ij/). Large condensates were determined as foci >50 ppixels or >100 ppixels in size, depending on the detection efficiency in a given experiment.

### RNAscope-ISH analysis

Cells cultured on coverslips were fixed in 10% neutral-buffered formalin, dehydrated through ascending ethanol concentrations, and kept in 100% ethanol at −20°C until use. On the day of assay, coverslips were rehydrated, washed with PBS, subjected to protease IV (ACDBio) pretreatment for 15 min at RT, washed again, and incubated with a custom FUS intron 6 RNAscope probe for 2 hours at 40°C. The probe was designed and manufactured by ACDBio/Bio-Techne (design #NPR-0039133). Signal was detected using an RNAscope 2.5 HD Assay–BROWN kit according to the manufacturer’s instructions. Coverslips were mounted for imaging using Immu-Mount (Thermo Fisher Scientific). Images were taken using either Olympus BX57 with an ORCA-Flash 4.0 camera or Nikon Eclipse Ni equipped with a Nikon DS-Ri1 camera.

### HyPro-MS analysis of the FUSint6&7-RNA condensate composition

Digoxigenin (DIG)-labeled DNA probe sets specific for FUSint6&7 were designed using the Stellaris online tool and custom-made by Eurofins (table S5). Probes were labeled using the second-generation DIG Oligonucleotide 3′ End Labeling Kit (Sigma-Aldrich) to yield 5 μM DIG-labeled mixtures. A modified recombinant HyPro enzyme was prepared, and HyPro labeling was performed in principle as described earlier (*38*). Briefly, cells grown in 10-cm dishes or on coverslips were fixed with dithiobis(succinimidyl propionate) (0.5 mg/ml; Thermo Fisher Scientific) in 1× PBS for 30 min at RT. The samples were then washed with 1× PBS/20 mM tris-HCl (pH 8.0), permeabilized with 70% ethanol, equilibrated in 2× SSC and 10% formamide (Thermo Fisher Scientific), and hybridized with DIG-labeled probes (100 nM for FUSint6&7 and 150 nm for ACTB) in 2× SSC, 10% formamide, and 10% dextran sulfate overnight at 37°C. Samples were washed with 10% formamide in 2× SSC and at 37°C for 30 min and 1× SSC at RT for 15 min and blocked with 0.8% bovine serum albumin (BSA) in 4× SSC (HyPro blocking buffer) treated with 100 murine RNase inhibitor (New England Biolabs). Samples were incubated with a modified HyPro enzyme (2.7 μg/ml) in HyPro blocking buffer at RT for 1 hour. After washing off unbound HyPro, proximity biotinylation was carried out in the presence of 0.5 mM biotin-phenol (Caltag Medsystems) and 0.1 mM hydrogen peroxide (Sigma-Aldrich). The reaction was quenched by 5 mM Trolox (Sigma-Aldrich) and 10 mM sodium ascorbate (Sigma-Aldrich, catalog no. A4034) in 1× PBS. Samples labeled in dishes were then analyzed by immunoblotting, mass spectrometry, or qRT-PCR. The coverslips were used for HyPro-FISH. Cells on dishes were lysed in RIPA buffer with 10 mM sodium ascorbate, 5 mM Trolox, 50 mM dithiothreitol (DTT), cOmplete, EDTA-free protease inhibitor cocktail (Sigma-Aldrich), and 1 mM phenylmethylsulfonyl fluoride (Cell Signaling), sonicated, de-crosslinked, and cleared by centrifugation. MyOne streptavidin C1 magnetic beads were incubated with de-crosslinked lysates for 1 hour at RT. The beads were washed twice with RIPA buffer, once with 1 M KCl, once with 0.1 M Na_2_CO_3_, once with 2 M urea in 10 mM tris-HCl, (pH 8.0), and twice with RIPA buffer. The beads were collected using the DynaMag-2 magnet and analyzed by mass spectrometry performed by the CEMS Proteomics Core Facilities at King’s College London as described (*38*). Raw mass-spec data files were processed using Proteome Discoverer (v2.2; Thermo Fisher Scientific) to search against UniProt SwissProt *Homo sapiens* taxonomy (49,974 entries) using Mascot (v2.6.0; www.matrixscience.com) and the Sequest search algorithms. Precursor mass tolerance was set to 20 parts per million with fragment mass tolerance set to 0.8 Da with a maximum of two missed cleavages. Variable modifications included carbamidomethylation (Cys) and oxidation (Met). Searching stringency was set to 1% false discovery rate (FDR). In total, 2869 proteins were detected. Label-Free Quantification (LFQ) intensity output was filtered against proteins commonly found in the proteomic contamination database CRAPome (*39*). Keratins were also removed. The filtered data were then then imported to R and analyzed using the DEP package (https://bioconductor.org/packages/release/bioc/vignettes/DEP/inst/doc/DEP.html). The data were filtered to include the proteins only identified in all three replicates, with default imputation settings (fun = “MinProb,” *q* = 0.01). DEP-generated *P* values were adjusted for multiple testing using the Benjamini-Hochberg (FDR) method. Raw data are available via the PRIDE (*79*)—ProteomeXchange (PXD063191).

### RNA purification and qRT-PCR

RNA was extracted either from total cell lysates or from nuclear and cytoplasmic fractions using TRI-reagent (Sigma-Aldrich) with a heating step (55°C for 10 min). In semiextractability analysis, nonheated samples (kept at RT) were analyzed in parallel (*17*). First-strand cDNA synthesis was performed using 500 ng of RNA with random primers (Promega) and MMLV reverse transcriptase (Promega) as per the manufacturer’s protocol (25 μl of final reaction volume). qRT-PCR was run using qPCRBIO SyGreen Lo-ROX mix on the CFX96/C1000 qPCR system (Bio-Rad). Samples were analyzed in at least two technical repeats, and expression of specific genes was determined using the 2^–∆∆Ct^ method and GAPDH for normalization. Primer sequences are given in table S4. Primers were designed using the National Center for Biotechnology Information Primer design tool (BLAST+Primer3).

### Western blotting

Total cell lysates were prepared by adding 2× Laemmli buffer directly to the wells in a 24-well plate followed by denaturation at 95°C for 10 min. SDS–PAGE and detection of proteins were carried out as described before (*52*). The following commercial primary antibodies were used (1:1000 dilution): FUS (rabbit polyclonal, Proteintech, 11570-1-AP, RRID:AB_2247082 or rabbit polyclonal, Bethyl, A300-293, RRID:AB_263409) and YTHDC1 (rabbit polyclonal, Proteintech, 29441-1-AP). Membranes were imaged on an Odyssey FC imaging system (700-nm channel), with band intensity quantified on Image Studio (LICORbio).

### Analysis of total m6A-RNA levels

For dot blot, 500 ng of total RNA was UV-crosslinked to Hybond-N+ membrane (Merck), which was subsequently probed with an m6A-specific antibody (mouse monoclonal, Proteintech, 68055-1-Ig, RRID:AB_2918796) as described for western blot. Results were validated using an independent antibody (rabbit polyclonal, Cell Signaling, 56593, RRID:AB_2799515). The EpiQuik m6A RNA Methylation Quantification Kit (EpigeneTek, P-9005) was used on the same set of RNA samples (200 ng of RNA per well) with inclusion of additional controls (FUS KO and FUS-GFP–expressing cells), according to the manufacturer’s instructions.

### RNA immunoprecipitation

WT and FUSΔNLS SH-SY5Y cells were transfected with pcDNA3-FLAG-HA-hYTHDC1 plasmid (gift from S. Jaffrey, Addgene plasmid #85167) (*80*) or control plasmid (Venus-Flag, made in-house) and harvested 24 hours posttransfection in the IP buffer (1× PBS and 1% Triton X-100 with cOmplete Mini Protease Inhibitor Cocktail, Roche). Transfection efficiency in all cell lines was verified by western blot. After a 30-min lysis on ice with periodic vortexing, the lysates were cleared by centrifuging at 13,000 rpm for 20 min. DYKDDDDK Fab-Trap Agarose beads (ChromoTek) were added to each supernatant sample and incubated for 3 hours on a nutator at 4°C. Beads were washed with IP buffer three times, and bound RNAs were eluted in TRI-reagent. RNA purification, cDNA synthesis and qRT-PCR were performed as above, using 150 ng of RNA as a template. RNA levels were normalized to GAPDH mRNA. The FUSint6&7-RNA level was normalized to GAPDH and then to the total FUSint6&7-RNA level in the respective cell line.

### Methylated RNA immunoprecipitation

Total RNA was isolated from WT and FUSΔNLS SH-SY5Y cells using TRI-reagent with heating. For immunoprecipitation, 6 μg of a mouse anti-m6A antibody (mouse monoclonal, Proteintech, 68055-1-Ig, RRID:AB_2918796) was incubated with 25-μl Dynabeads Protein G (Invitrogen, 10003D) in IP buffer [10 mM tris (pH 7.5), 150 mM NaCl, and 0.1% NP-40] for 1 hour at 4°C, followed by washes with the same buffer. Subsequently, 30 μg of total RNA was added to the m6A antibody–coated beads for 2 hours at 4°C. Uncoated beads were used as a control. After IP buffer washes, the beads were resuspended in TRI-reagent, followed by RNA isolation. cDNA synthesis and qPCR were performed as above, using 170 ng of RNA as a template. The methylated FUSint6&7-RNA level was normalized to GAPDH mRNA (extensively methylated transcript), then to the total FUSint6&7-RNA level in the respective cell line, and lastly, to no-antibody beads control.

### Analysis of m6A effect on FUS condensation in vitro by ImmuCon

ImmuCon Assay is described in detail in (*49*). Recombinant FUS (produced in-house; 2.5 μM final concentration) was mixed with 250 nM unmodified or m6A-modified 4XDRACH RNA oligonucleotide in assay buffer [20 mM tris-HCl (pH 7.5), 20 mM KCl, 2 mM MgCl_2_, and 1 mM DTT]. RNA oligonucleotides (5′-end labeled with Cy5 and 3′-end labeled with biotin-TEG) were custom-made and high-performance liquid chromatography–purified by Horizon (* denotes m6A-modified adenosine): 5′-GGACUCGGACUUGGACUCUGGACUUUG-3′ and 5′-GGA*CUCGGA*CUUGGA*CUCUGGA*CUUUG-3′. A microdroplet (5 μl) of each sample was placed on a 10-mm round glass coverslip; condensates were left to sediment for 15 min and then fixed with 2% glutaraldehyde in PBS for 15 min. The coverslips were blocked in 1% BSA in PBS for 1 hour at RT and subsequently incubated with an anti-FUS antibody (1:5000; rabbit polyclonal, Proteintech, 11570-1-AP, RRID:AB_2247082) for 2 hours at RT. After PBS washes, an anti-rabbit Alexa Fluor 546 antibody (1:1000; Invitrogen) was applied for 1 hour. The coverslips were mounted with Immu-Mount (Thermo Fisher Scientific), imaged using an Olympus BX57 upright microscope and an ORCA-Flash 4.0 camera, and processed using the cellSens Dimension software (Olympus). The FUS condensate area was quantified using the Analyze Particles tool in ImageJ.

### Confocal nanoscanning

CONA assay was performed as described for TDP-43 (*52*) with modifications. Plasmid for YTHDF1-GFP expression was a gift from E. Izaurralde (pT7-EGFP-C1-HsYTHDF1_AB, Addgene plasmid #148307). Briefly, HeLa cells transfected to express either GFP alone or GFP-tagged FUS or YTHDF1 in 35-mm dishes were lysed in 500 μl of 1% Triton X-100/PBS with RiboLock (40 U/ml; Thermo Fisher Scientific) for 15 min on ice with periodic vortexing. Lysates were cleared by centrifuging at 13,000 rpm for 15 min, snap-frozen, and kept in −80°C. Ni-NTA (nickel-nitrilotriacetic acid) Superflow beads (QIAGEN) were washed with binding buffer [20 mM Hepes (pH 7.5), 0.3 M NaCl, 0.01% Triton X-100, and 5 mM MgCl_2_], coated with His-streptavidin (NKMAX), and then with the respective RNA oligonucleotide described above—4xDRACH or 4xDRACH-m6A (50 pmol) or “generic” RNA oligonucleotide mix [a mix of (UG)_15_, (AUG)_12_, and Clip34nt, in equal ratios]. Beads were washed in binding buffer and added to the thawed cell lysates containing the GFP-tagged protein and incubated on a multiorbital rotator for 2 hours at RT. After washes in binding buffer, beads were imaged in a μCLEAR 384-well plate (Greiner) on Opera Phenix. Mean ring intensity for Cy5 and EGFP channels was quantified using a custom automated pipeline on Harmony 4.9.

### Electrophoretic mobility shift assay

Cy5-labeled RNA oligonucleotides 4xDRACH and 4xDRACH-m6A were incubated at 250 nM with recombinant FUS (as above; at 0, 2.5, 5.0, and 7.0 μM) or YTHDF1 (Active Motif #31608; at 0.64 μM) in the assay buffer [50 mM tris-HCl (pH 7.5), 100 mM KCl, 2 mM MgCl_2_, 100 mM β-mercaptoethanol, and BSA (0.1 mg/ml)] for 30 min at RT with gentle shaking. Samples were analyzed on 6% native acrylamide gel in tris-boric acid–EDTA buffer, followed by imaging on an Odyssey FC imaging system (700-nm channel) and band intensity quantification on Image Studio (LICORbio).

### Data visualization and statistics

Processed data visualization and statistical analyses were performed using GraphPad Prism or R, unless indicated otherwise. Mean values of replicates were compared using appropriate statistical tests. Statistical tests used are indicated in figure legends with statistical significance denoted with asterisks: *P < 0.05, **P < 0.01, ***P < 0.001, and ****P < 0.0001. *N* indicates the number of biological replicates. Error bars represent SD unless indicated otherwise.
